# Supplementary material for: Electronic mechanism of sub-100-fs demagnetization induced by a femtosecond light pulse
Source: Sci Rep. 2026 May 11;16:14705. doi: 10.1038/s41598-026-51949-2 (PMC13161376; doi:10.1038/s41598-026-51949-2)
Supplement: Supplementary file 1 — Supplementary Information. [file 41598_2026_51949_MOESM1_ESM.pdf]

Supplementary Information

Electronic mechanism of sub-100-fs demagnetization induced by a femtosecond light pulse

Konrad J. Kapcia,[1,2] Victor Tkachenko,[3,2] Flavio Capotondi,[4]
Alexander Lichtenstein,[3,5] Serguei Molodtsov,[3,6,7] Przemysław Piekarz,[8] and Beata Ziaja[2,8]

[1] *Institute of Spintronics and Quantum Information, Faculty of Physics and Astronomy, Adam Mickiewicz University in Poznań, Uniwersytetu Poznańskiego 2, 61614 Poznań, Poland*
[2] *Center for Free-Electron Laser Science CFEL, Deutsches Elektronen-Synchrotron DESY, Notkestr. 85, 22607 Hamburg, Germany*
[3] *European XFEL GmbH, Holzkoppel 4, 22869 Schenefeld, Germany*
[4] *Elettra-Sincrotrone Trieste S.C.p.A, 34149 Trieste, Basovizza, Italy*
[5] *University of Hamburg, Jungiusstr. 9, 20355 Hamburg, Germany*
[6] *Institute of Experimental Physics, TU Bergakademie Freiberg, Leipziger Strasse 23, 09599 Freiberg, Germany*
[7] *Center for Efficient High Temperature Processes and Materials Conversion (ZeHS), TU Bergakademie Freiberg, Winklerstrasse 5, 09599 Freiberg, Germany*
[8] *Institute of Nuclear Physics, Polish Academy of Sciences, Radzikowskiego 152, 31-342 Kraków, Poland*

SUPPLEMENTARY METHODS

Modeling interaction of X-rays with solid materials using XSPIN code

Modeling radiation damage in solid materials has been performed for several years with various simulation techniques, e.g., [1–4]. Among others, our in-house hybrid code XTANT (X-ray-induced Thermal And Nonthermal Transitions) [1, 5–9] was developed. It uses periodic boundary conditions for the simulations of X-ray irradiated bulk materials, enabling computationally inexpensive simulations of relatively large supercells (containing up to a few thousands atoms), The code includes a few modules dedicated to simulate microscopic process triggered by the incoming X-ray FEL radiation, also out of equilibrium. They are listed below.

(a) Band structure module (in refs. [1, 5–9] based on transferable tight binding Hamiltonian, in ref. [10] replaced by the DFTB+ code [11]) calculates the transient electronic band structure of low-energy electrons and the transient potential energy surface. The latter is used to calculate the actual forces acting on nuclei, depending on their positions in the simulation box. Electron occupation numbers, distributed on the transient energy levels in the band, are assumed to follow Fermi-Dirac distribution with a transient temperature and a transient chemical potential. The electronic parameters change due to the interaction of band electrons with X-rays and high-energy electrons.

(b) Classical molecular dynamics (MD) module models change of nuclei positions in time. It solves Newton equations for nuclei, with the potential energy surface evaluated from the band structure module.

(c) Classical event-by-event Monte Carlo (MC) simulation module treats fraction of high-energy electrons and Auger decays of core holes. It stochastically models X-ray induced photoelectron emission from deep shells or from the valence band, the Auger decays, and the scattering of high-energy electrons. In the code, at each time step an intrinsic averaging over 30000 different Monte Carlo realizations of electron (and core hole) trajectories is

performed to calculate the average electronic distribution which is then applied at the next time step.

The code XSPIN applies this scheme to magnetic materials, distinguishing between spin-up and spin-down electrons and introducing spin conservation in scattering processes. For more details, see [12] and [13–16].

Modeling of spin flip processes

The microscopic processes following X-ray irradiation on femtosecond timescales include: (i) photoionization, (ii) Auger decay, and (iii) impact (collisional) ionization. Photoionization can excite an inner- or outer-shell electron either from the spin-up or the spin-down electronic fraction. If an inner-shell excitation occurred, the remaining core hole relaxes via Auger decay. A band/core electron with the same spin then fills the hole, while an Auger electron is excited from the electronic subsystem (either a spin-up or a spin-down one), with the probability following the actual occupation of the spin-up and the spin-down domains. Concerning excited electrons, the XSPIN tool distinguishes between high energy electrons (HEF), with energy above some fixed threshold, and low energy electrons (LEF), with energy below some fixed threshold. The HEF electrons are treated as particles (with Monte Carlo method). They may also excite further electrons with the same or an opposite spin in collisional processes. The probability of such an excitation depends on the actual occupations of the spin-up and spin-down electron levels in the LEF and in the core shells. During the sequence of the following impact ionization events, the HEF electron continuously loses its energy and may ultimately fall into the spin-up or the spin-down subsystem of the LEF, depending on its spin state.

In the LEF, the electron occupation numbers are distributed on transient energy levels, folllowing an assumed transient Fermi-Dirac distribution with a transient temperature and transient chemical potential. Both temperature and chemical potential have to be adjusted at each simulation time step ($\Delta t \sim 0.01$ fs) as the total electronic occupation and the energy of the LEF changes with time. This is due to the on-going excitations of LEF electrons from bands (through photoinduced or collisional processes) and the relaxation of high-energy electrons to the LEF. Note that in our model a spatial distribution of electrons does not play a role, as the periodic boundary condition framework is applied in the simulations.

At each time step, we enforce a common (new) transient electronic temperature and a common (new) transient chemical potential for the entire electronic system, i.e., for electron fractions both with spin up and with spin down. This process reorganizes transient electronic occupations both in the spin-up and the spin-down domains of the LEF at each simulation time step. Any change of the electronic temperature must then induce a migration of electrons between the spin-up and spin-down fractions, accounting for spin flip processes (i.e., the Hund exchange). As the occupations of spin-up and spin-down electron fractions change with time, this way the total transient magnetization of material changes with time, because it is calculated from the transient occupations of spin-up and spin-down electronic levels at each simulation time step.

SUPPLEMENTARY RESULTS

In Supplementary Table S1 we provide the estimation of the fluence for the bulk systems (with sample thickness equal or larger than the attenuation length) for all cases we have studied in the manuscript (based on the data from [17–19]). For optical and soft X-ray photons used in the work, absorbed doses corresponds to the beam fluence approximately between 20 and 30 mJ/cm^2. The fluence estimates are obtained using the Beer–Lambert law applied to bulk materials, assuming that the sample thickness is equal to the attenuation length of the material.

TABLE S1. Calculated values of beam fluence F (in mJ/cm^2) corresponding to the average absorbed dose of 0.93 eV/atom for different photon energies $\hbar\omega$ (in eV). Symbol λ denotes photon attenuation length (in nm).

$\hbar\omega$ [eV]	λ [nm]	F [mJ/cm^2]	$\hbar\omega$ [eV]	λ [nm]	F [mJ/cm^2]
	Cobalt			Nickel	
2.0	12.0	25.8	2.0	12.2	26.3
61.1	9.2	19.7	67.3	15.2	32.7
1000.0	115.6	247.9	1000.0	107.6	231.7
10000.0	6189.8	13268.0	10000.0	5379.5	11587.4

Electronic density of states

The electronic density of states (DOS) $D_\sigma(\varepsilon)$ for the studied systems was calculated using the Vienna Ab-initio Simulation Package (VASP) [20–23]. In VASP, projector augmented-wave (PAW) potentials and the generalized gradient approximation (GGA) [24] in the Pardew, Burke, and Ernzerhof (PBE) parameterization [25] are used to calculate the electronic density of states for bulk fcc Co and fcc Ni. Nine valence electrons for Co atoms ($3d^7$, $4s^2$) and ten electrons for Ni atoms ($3d^8$, $4s^2$) are taken. An energy cutoff for the plane-wave expansion of 300 eV and 270 eV was applied, respectively, and a Γ-centered mesh of $27 \times 27 \times 27$ points was used in the primitive cell (containing one atom). The resulting spin-resolved DOS for both materials are presented in Supplementary Fig. S1 (DOS for minority spins is presented as negative). The ground state (GS, at $T = 0$ K) magnetization (per atom) resulting from ab initio calculations is $m_{\rm GS}^{\rm Co} = 1.63$ and $m_{\rm GS}^{\rm Ni} = 0.62$ for cobalt and nickel, respectively.

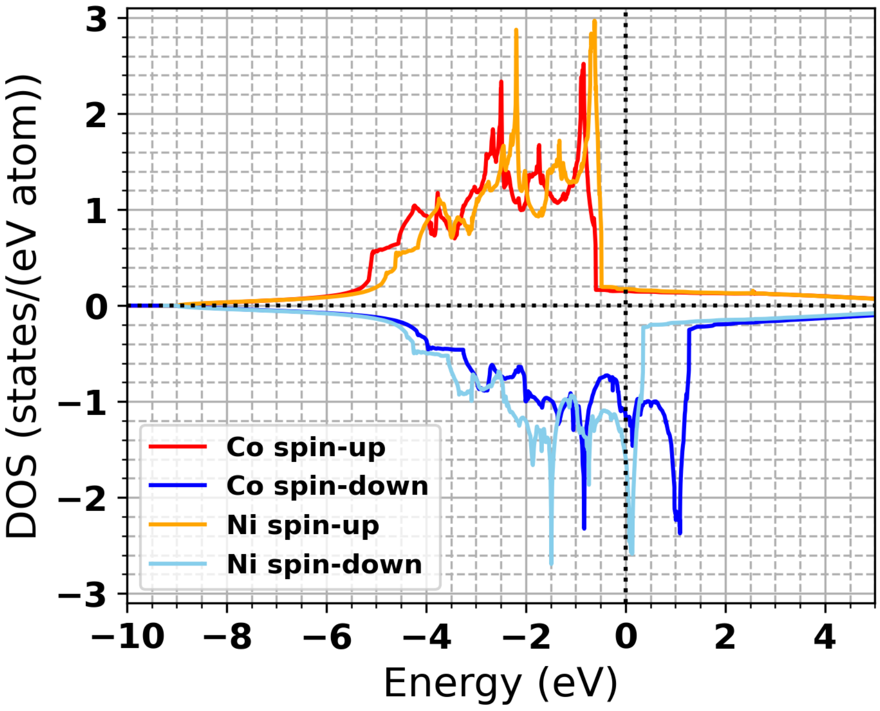

FIG. S1. The calculated ab-initio spin-resolved densities of states (states per eV per atom) for fcc cobalt (red and blue lines) and fcc nickel (orange and light-blue lines), obtained using VASP. The minority-spin DOS is plotted with negative values for clarity. The Fermi level (defined as the highest occupied state at the ground state) is located at zero energy.

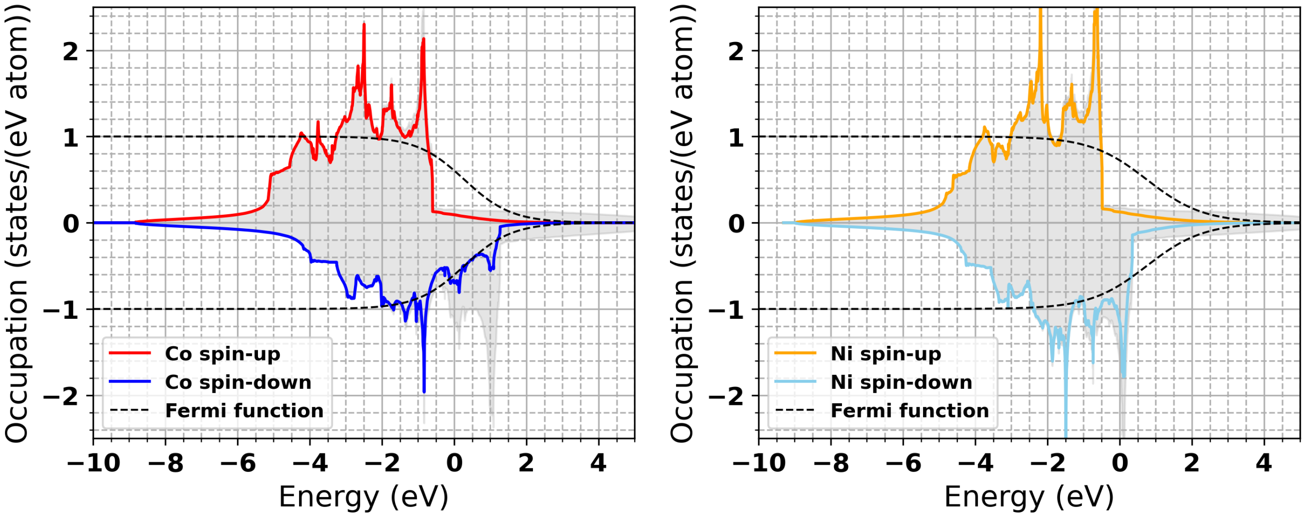

FIG. S2. The electronic density of states for low-energy electrons including occupations for cobalt (left panel) and nickel (right panel) after the end of the pulse (i.e., after 75 fs). For clarity, the values corresponding to minority-spin electrons are plotted with negative sign. The black lines represent the Fermi–Dirac distribution computed for the obtained effective electronic parameters, whereas the grey shadings indicate the density of states taken from Supplementary Fig. S1 for comparison.

To illustrate the effects of incoming pulse, we introduce a quantity $\bar{D}_\sigma(\varepsilon) = D_\sigma(\varepsilon) f(\varepsilon)$ (the DOS including the occupations) as product of the electronic DOS $D_\sigma(\varepsilon)$ and the Fermi-Dirac distribution $f(\varepsilon) = 1/\left\{1 + \exp\left[(\varepsilon - \mu)/k_\mathrm{B} T\right]\right\}$, where μ and T are the effective parameters (chemical potential and temperature) describing low-energy electrons found within our model. The visualizations of the DOSs with the occupation $\bar{D}_\sigma(\varepsilon)$ after the end of the pulse (i.e., after 75 fs) for both materials studied are presented in Supplementary Fig. S2. In the figure, we used the following effective parameters resulting from the simulations by the XSPIN for 2 eV photons and a pulse of 70 fs FWHM duration: $\mu = 0.27$ eV and $T = 7463$ K for cobalt; $\mu = 0.753$ eV and $T = 9210$ K for nickel.

Electron excitation and magnetic parameters for additional incoming photon energies

We also present a comparison of electron excitation and magnetic parameters in the irradiated single domain of Co for the incoming photon energies of ~ 61 eV (above Co M-edge) and 10000 eV (Supplementary Figs. S3 and S4). The observations are similar to those made for the 2 eV vs. 1000 eV case. For a pulse duration of 70 fs FWHM, there is practically no difference in the predicted temporal characteristics of core hole populations, electronic excitation and magnetization in a single magnetic domain for both energy cases. However, for the 2 fs FWHM pulse, the difference between the 61 eV and the 10000 eV cases is visible. This is due to the long electron cascading time for electrons released by a 10000 eV photon impact (9.8 fs; cascading time is 4.0 fs for 1000 eV photons). In contrast, after femtosecond optical pumping, temporal changes of electronic and magnetic parameters in a single domain in Co are determined almost exclusively by the temporal pulse shape. They proceed without delays due to the absence of electronic cascading.

We also include similar results for Ni for a photon energy close to the M-edge of Ni (67.3 eV) and for 10000 eV X-ray photons (Supplementary Figs. S5 and S6). The electron cascading times for Ni are: 3.9 fs for 1000 eV and 10.0 fs for 10000 eV.

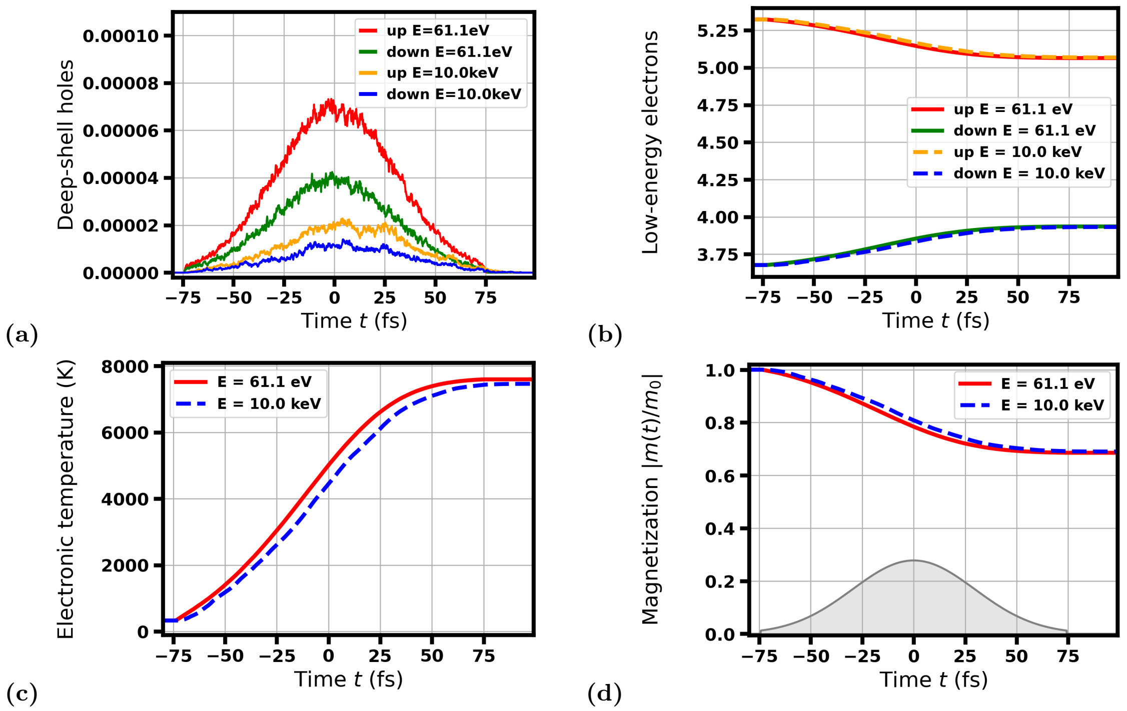

FIG. S3. Transient electronic and magnetic properties of Co predicted by XSPIN in a single magnetic domain under X-ray irradiation with $\sim$ 61 eV pulse or X-ray irradiation with 10000 eV pulse (as labeled), both of 70 fs FWHM duration: (a) number of deep-shell holes produced per atom (spin-resolved), (b) number of low-energy electrons per atom (with the energy below the cutoff of 15 eV, spin-resolved), (c) electronic temperature, (d) magnetization (normalized to its initial value m_0 before the pulse), all plotted as a function of time. Temporal profile of the pulse triggering the dynamics is schematically depicted.

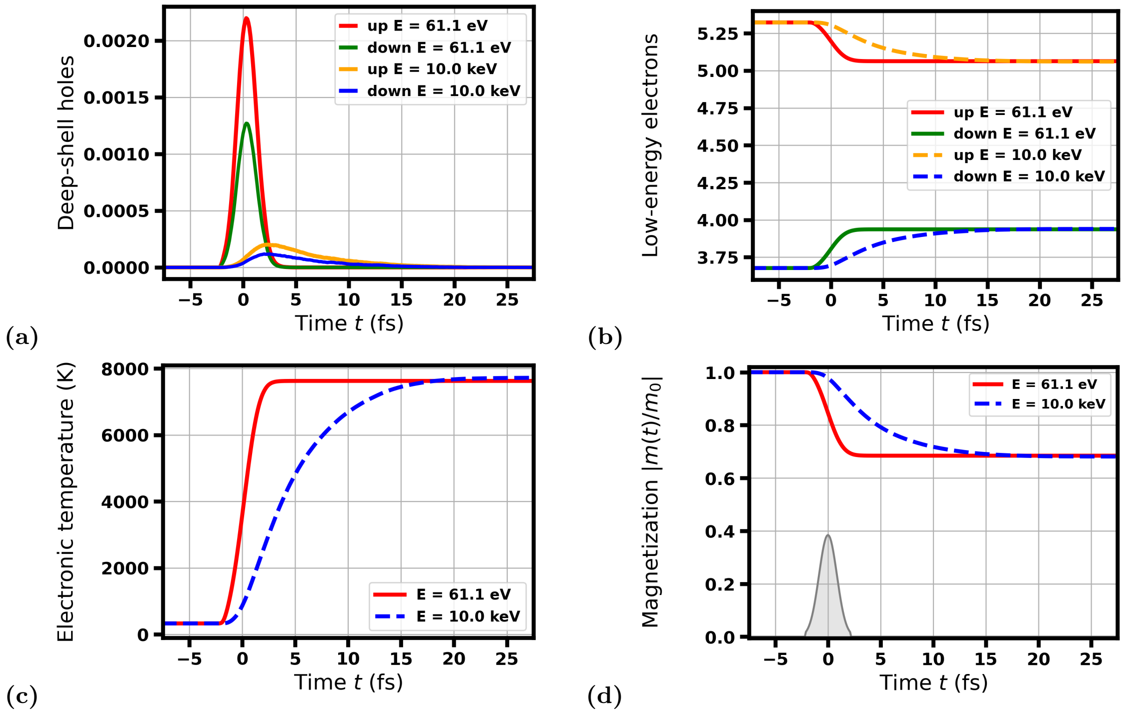

FIG. S4. Transient electronic and magnetic properties of Co predicted by XSPIN in a single magnetic domain under X-ray irradiation with $\sim$ 61 eV pulse or X-ray irradiation with 10000 eV pulse (as labeled), both of 2 fs FWHM duration. Other simulation parameters are as in Fig. S3.

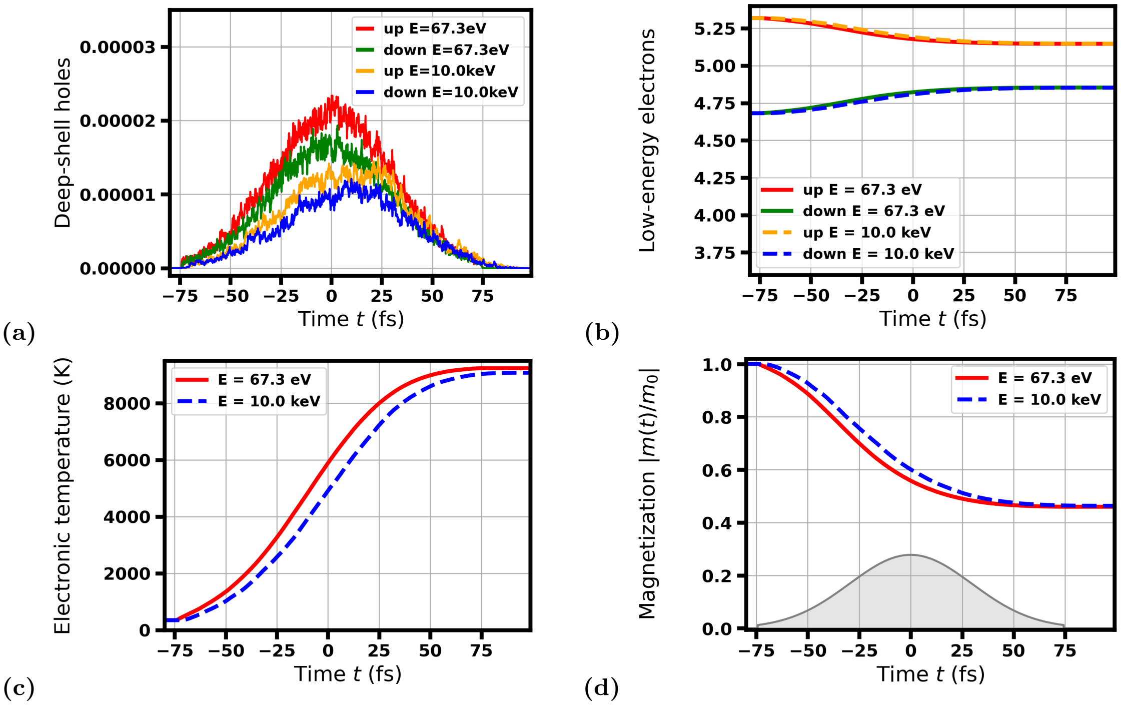

FIG. S5. Transient electronic and magnetic properties of Ni predicted by XSPIN in a single magnetic domain under X-ray irradiation with ∼ 67 eV pulse or X-ray irradiation with 10000 eV pulse (as labeled) of 70 fs FWHM duration. Other simulation parameters are as in Fig. S3.

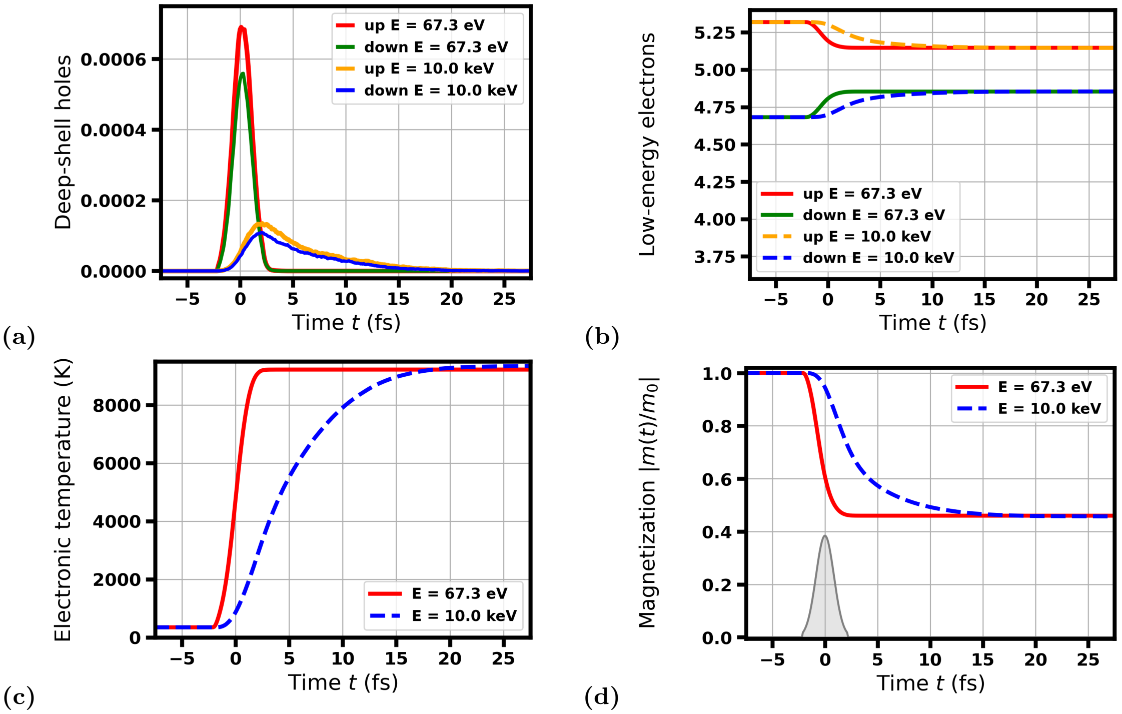

FIG. S6. Transient electronic and magnetic properties of Ni predicted by XSPIN in a single magnetic domain under X-ray irradiation with ∼ 67 eV pulse or X-ray irradiation with 10000 eV pulse (as labeled) of 2 fs FWHM duration. Other simulation parameters are as in Fig. S3.

SUPPLEMENTARY LITERATURE

Note that all references quoted above in Supplementary Information and listed below are also cited in the main text of the manuscript, however, with different numbers.

---

[1] N. Medvedev, H. O. Jeschke, and B. Ziaja, Nonthermal phase transitions in semiconductors induced by a femtosecond extreme ultraviolet laser pulse, New J. Phys. **15**, 015016 (2013).

[2] B. F. Murphy, T. Osipov, Z. Jurek, L. Fang, S. K. Son, M. Mucke, J. H. D. Eland, V. Zhaunerchyk, R. Feifel, L. Avaldi, P. Bolognesi, C. Bostedt, J. D. Bozek, J. Grilj, M. Guehr, L. J. Frasinski, J. Glownia, D. T. Ha, K. Hoffmann, E. Kukk, B. K. McFarland, C. Miron, E. Sistrunk, R. J. Squibb, K. Ueda, R. Santra, and N. Berrah, Femtosecond x-ray-induced explosion of C60 at extreme intensity, Nat. Commun. **5**, 4281 (2014).

[3] K. R. Beyerlein, H. O. Jönsson, R. Alonso-Mori, A. Aquila, S. Bajt, A. Barty, R. Bean, J. E. Koglin, M. Messerschmidt, D. Ragazzon, D. Sokaras, G. J. Williams, S. Hau-Riege, S. Boutet, H. N. Chapman, N. Tîmneanu, and C. Caleman, Ultrafast nonthermal heating of water initiated by an x-ray free-electron laser, PNAS **115**, 5652 (2018).

[4] P. J. Ho, B. J. Daurer, M. F. Hantke, J. Bielecki, A. Al Haddad, M. Bucher, G. Doumy, K. R. Ferguson, L. Flückiger, T. Gorkhover, B. Iwan, C. Knight, S. Moeller, T. Osipov, D. Ray, S. H. Southworth, M. Svenda, N. Timneanu, A. Ulmer, P. Walter, J. Hajdu, L. Young, F. R. N. C. Maia, and C. Bostedt, The role of transient resonances for ultra-fast imaging of single sucrose nanoclusters, Nat. Commun. **11**, 167 (2020).

[5] N. Medvedev, H. O. Jeschke, and B. Ziaja, Nonthermal graphitization of diamond induced by a femtosecond x-ray laser pulse, Phys. Rev. B **88**, 224304 (2013).

[6] V. Tkachenko, N. Medvedev, Z. Li, P. Piekarz, and B. Ziaja, Transient optical properties of semiconductors under femtosecond x-ray irradiation, Phys. Rev. B **93**, 144101 (2016).

[7] N. Medvedev, V. Tkachenko, V. Lipp, Z. Li, and B. Ziaja, Various damage mechanisms in carbon and silicon materials under femtosecond x-ray irradiation, 4open **1**, 3 (2018).

[8] N. Medvedev and B. Ziaja, Multistep transition of diamond to warm dense matter state revealed by femtosecond x-ray diffraction, Sci. Rep. **8**, 5284 (2018).

[9] N. Medvedev, Z. Li, V. Tkachenko, and B. Ziaja, Electron-ion coupling in semiconductors beyond Fermi's golden rule, Phys. Rev. B **95**, 014309 (2017).

[10] V. Lipp, V. Tkachenko, M. Stransky, B. Aradi, T. Frauenheim, and B. Ziaja, Density functional tight binding approach utilized to study x-ray-induced transitions in solid materials, Sci. Rep. **12**, 1551 (2022).

[11] B. Hourahine, B. Aradi, V. Blum, F. Bonafé, A. Buccheri, C. Camacho, C. Cevallos, M. Y. Deshaye, T. Dumitrică, A. Dominguez, S. Ehlert, M. Elstner, T. van der Heide, J. Hermann, S. Irle, J. J. Kranz, C. Koehler, T. Kowalczyk, T. Kubař, I. S. Lee, V. Lutsker, R. J. Maurer, S. K. Min, I. Mitchell, C. Negre, T. A. Niehaus, A. M. N. Niklasson, A. J. Page, A. Pecchia, G. Penazzi, M. P. Persson, J. Řezáč, C. G. Sánchez, M. Sternberg, M. Stoehr, F. Stuckenberg, A. Tkatchenko, V. W. z. Yu, and T. Frauenheim, DFTB+, a software package for efficient approximate density functional theory based atomistic simulations, J. Chem. Phys. **152**, 124101 (2020).

[12] K. J. Kapcia, V. Tkachenko, F. Capotondi, A. Lichtenstein, S. Molodtsov, L. Müller, A. Philippi-Kobs, P. Piekarz, and B. Ziaja, Modeling of ultrafast x-ray induced magnetization dynamics in magnetic multilayer systems, npj Comput. Mater. **8**, 212 (2022).

[13] K. J. Kapcia, V. Tkachenko, F. Capotondi, A. Lichtenstein, S. Molodtsov, L. Mueller, A. Philippi-Kobs, P. Piekarz, and B. Ziaja, Electronic processes occurring during ultrafast demagnetization of cobalt triggered by x-ray photons tuned to the Co L_3 resonance, Phys. Rev. B **107**, 094402 (2023).

[14] K. J. Kapcia, V. Tkachenko, F. Capotondi, A. Lichtenstein, S. Molodtsov, P. Piekarz, and B. Ziaja, Ultrafast demagnetization in bulk nickel induced by x-ray photons tuned to Ni M_3 and L_3 absorption edges, Sci. Rep. **14**, 473 (2024).

[15] K. J. Kapcia, V. Lipp, V. Tkachenko, and B. Ziaja, Theoretical analysis of x-ray free-electron-laser experimental data using Monte-Carlo and molecular-dynamics based computational tools, in *Comprehensive Computational Chemistry*, Vol. 3, edited by M. Yáñez and R. J. Boyd (Elsevier, Oxford, 2024) 1st ed., pp. 858–864.

[16] S. Antunes, K. J. Kapcia, V. Lipp, and B. Ziaja, Ultrafast transitions in x-ray irradiated solids, Contributions to Plasma Physics **n/a**, e70095 (2026).

[17] A. Y. C. Yu, T. M. Donovan, and W. E. Spicer, Optical properties of cobalt, Phys. Rev. **167**, 670 (1968).

[18] P. B. Johnson and R. W. Christy, Optical constants of transition metals: Ti, V, Cr, Mn, Fe, Co, Ni, and Pd, Phys. Rev. B **9**, 5056 (1974).

[19] B. L. Henke, E. M. Gullikson, and J. C. Davis, X-ray interactions: photoabsorption, scattering, transmission, and reflection at $E = 50 - 30000$ eV, $Z = 1 - 92$, Atomic Data and Nuclear Data Tables **54**, 181 (1993).

[20] The Vienna Ab initio Simulation Package: Atomic scale materials modelling from first principles, https://www.vasp.at .

[21] G. Kresse and J. Hafner, Ab initio molecular-dynamics simulation of the liquid-metal–amorphous-semiconductor transition in germanium, Phys. Rev. B **49**, 14251 (1994).

[22] G. Kresse and J. Furthmüller, Efficient iterative schemes for ab initio total-energy calculations using a plane-wave basis set, Phys. Rev. B **54**, 11169 (1996).

[23] G. Kresse and D. Joubert, From ultrasoft pseudopotentials to the projector augmented-wave method, Phys. Rev. B **59**, 1758 (1999).

[24] P. E. Blöchl, Projector augmented-wave method, Phys. Rev. B **50**, 17953 (1994).

[25] J. P. Perdew, K. Burke, and M. Ernzerhof, Generalized gradient approximation made simple, Phys. Rev. Lett. **77**, 3865 (1996).
